# Prescribed Drug Use and Aneurysmal Subarachnoid Hemorrhage Incidence: A Drug-Wide Association Study

**DOI:** 10.1212/WNL.0000000000209479

**Published:** 2024-06-05

**Authors:** Jos P. Kanning, Shahab Abtahi, Christian Schnier, Olaf H. Klungel, Mirjam I. Geerlings, Ynte M. Ruigrok

**Affiliations:** From the UMC Utrecht Brain Center (J.P.K., Y.M.R.), Department of Neurology and Neurosurgery, University Medical Center Utrecht; Julius Center for Health Sciences and Primary Care (J.P.K., O.H.K., M.I.G.), University Medical Center Utrecht, and Division of Pharmacoepidemiology and Clinical Pharmacology (S.A., O.H.K.), Utrecht Institute for Pharmaceutical Sciences, Utrecht University, the Netherlands; Infection Medicine (C.S.), Edinburgh Medical School, The University of Edinburgh, United Kingdom; Department of General Practice (M.I.G.), Amsterdam UMC, location University of Amsterdam; Amsterdam Public Health, Aging & Later Life, and Personalized Medicine (M.I.G.); and Amsterdam Neuroscience, Neurodegeneration, and Mood, Anxiety, Psychosis, Stress, and Sleep (M.I.G.), the Netherlands.

## Abstract

**Background and Objectives:**

Current benefits of invasive intracranial aneurysm treatment to prevent aneurysmal subarachnoid hemorrhage (aSAH) rarely outweigh treatment risks. Most intracranial aneurysms thus remain untreated. Commonly prescribed drugs reducing aSAH incidence may provide leads for drug repurposing. We performed a drug-wide association study (DWAS) to systematically investigate the association between commonly prescribed drugs and aSAH incidence.

**Methods:**

We defined all aSAH cases between 2000 and 2020 using *International Classification of Diseases* codes from the Secure Anonymised Information Linkage databank. Each case was matched with 9 controls based on age, sex, and year of database entry. We investigated commonly prescribed drugs (>2% in study population) and defined 3 exposure windows relative to the most recent prescription before index date (i.e., occurrence of aSAH): current (within 3 months), recent (3–12 months), and past (>12 months). A logistic regression model was fitted to compare drug use across these exposure windows vs never use, controlling for age, sex, known aSAH risk factors, and health care utilization. The family-wise error rate was kept at *p* < 0.05 through Bonferroni correction.

**Results:**

We investigated exposure to 205 commonly prescribed drugs between 4,879 aSAH cases (mean age 61.4, 61.2% women) and 43,911 matched controls. We found similar trends for lisinopril and amlodipine, with a decreased aSAH risk for current use (lisinopril odds ratio [OR] 0.63, 95% CI 0.44–0.90, amlodipine OR 0.82, 95% CI 0.65–1.04) and an increased aSAH risk for recent use (lisinopril OR 1.30, 95% CI 0.61–2.78, amlodipine OR 1.61, 95% CI 1.04–2.48). A decreased aSAH risk in current use was also found for simvastatin (OR 0.78, 95% CI 0.64–0.96), metformin (OR 0.58, 95% CI 0.43–0.78), and tamsulosin (OR 0.55, 95% CI 0.32–0.93). By contrast, an increased aSAH risk was found for current use of warfarin (OR 1.35, 95% CI 1.02–1.79), venlafaxine (OR 1.67, 95% CI 1.01–2.75), prochlorperazine (OR 2.15, 95% CI 1.45–3.18), and co-codamol (OR 1.31, 95% CI 1.10–1.56).

**Discussion:**

We identified several drugs associated with aSAH, of which 5 drugs (lisinopril and possibly amlodipine, simvastatin, metformin, and tamsulosin) showed a decreased aSAH risk. Future research should build on these signals to further assess the effectiveness of these drugs in reducing aSAH incidence.

**Classification of Evidence:**

This study provides Class III evidence that some commonly prescribed drugs are associated with subsequent development of aSAH.

## Introduction

Aneurysmal subarachnoid hemorrhage (aSAH) is a type of stroke which occurs when an intracranial aneurysm ruptures, causing bleeding in the subarachnoid space.^1^ Despite the fact that aSAH only accounts for 10% of all strokes,^2^ the early age of onset and high mortality rate make the years of potential life lost because of aSAH comparable with those lost because of ischemic stroke, the most common type of stroke.^3^ In patients with known unruptured intracranial aneurysms, aSAH can be prevented by endovascular or neurosurgical treatment.^4^ However, these surgical treatment options carry a risk of permanent disability or mortality of up to 8%.^5,6^ As the potential benefits of these treatment options often do not outweigh the risk of treatment complications, most intracranial aneurysms remain untreated.^7^ Thus, a noninvasive drug compound that can prevent aneurysm rupture would be highly beneficial. Such drugs, however, have yet to be identified.

Conducting a drug-wide association study (DWAS) is a novel paradigm for drug discovery. A DWAS uses large electronic health care databases (EHDs) to generate hypotheses about the relationship between drugs and disease.^8^ Previous DWAS have effectively identified drug-outcome associations for diverse medical conditions, including myocardial infarction,^8^ cancer,^9,10^ dementia,^11,12^ and COVID-19.^13^ Signals derived from DWAS can be further investigated and validated, paving the way for potential drug repurposing.^14^

In this study, we conducted a hypothesis-generating DWAS using a large nationwide EHD. Our primary objective was to identify commonly prescribed drugs that were associated with a lower incidence of aSAH and could be used to prevent aneurysm rupture.

## Methods

### Setting

This study used data from the Secure Anonymised Information Linkage (SAIL) databank. The SAIL databank works with health care providers and government agencies to obtain a diverse array of anonymized data sets, and it currently includes anonymized, individual-level, linked routinely collected health care data (e.g., diagnoses, treatments, medical histories, hospital admissions and discharges, outpatient visits, and prescriptions) for approximately 80% of the population of Wales, the United Kingdom.^15^ For this investigation, we linked data from primary care (Welsh Longitudinal General Practice dataset), hospital admissions (Patient Episode Database for Wales), and mortality records (Annual District Death Extract). Primary care records in SAIL are cataloged using Read codes (version 2) while hospital admissions and mortality records use *International Classification of Diseases* versions 9 (*ICD-9*) and 10 (*ICD-10*) codes.

### Study Population and Outcome

**Figure 1:**
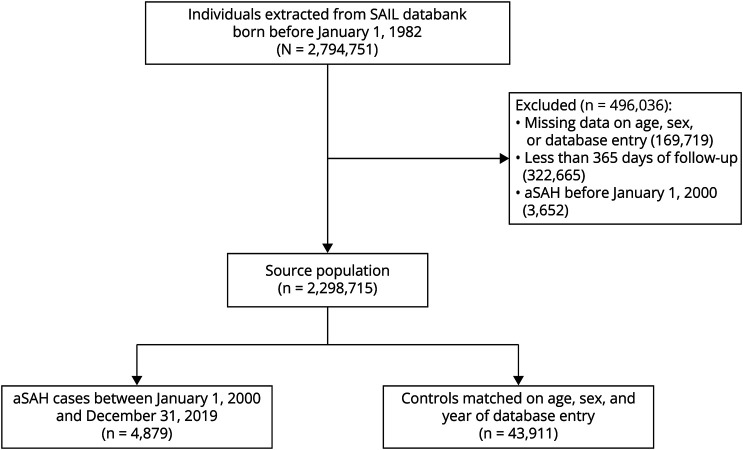
Study Flow Diagram aSAH = aneurysmal subarachnoid hemorrhage; SAIL = Secure Anonymised Information Linkage databank.

We included all patients born before January 1, 1982, with an aSAH hospital diagnosis (*ICD-9* code 430 and *ICD-10* codes I60.0–I60.9) and a date of hospital admission (from now on referred to as the index date) between January 1, 2000, and December 31, 2019 (Figure 1). Patients with an observation window shorter than 365 days or a record of aSAH (ICD codes above or Read codes Gyu61, Gyu60, Gyu6E, Gyu64) before January 2000 were excluded from further analysis. For each aSAH case, we randomly matched up to 9 controls without replacement based on year of birth and sex. In addition, we matched based on year of database entry to ensure comparable observation windows for cases and controls. Database entry was defined as whichever came first: January 1, 2000, or the first primary care record between January 2000 and December 2019. Similarly, database exit was defined as whichever came first: December 31, 2019, date of death, date of SAIL databank exit (e.g., due to migration), or date of aSAH diagnosis. Controls with a database exit before a case's diagnosis date were no longer eligible to be matched to that case. In addition, controls were required to have a minimum observation window of 365 days. Each matched control was given the same index date as the case they were matched with.

### Exposure Definition

In SAIL, primary care records are encoded using 5-digit Read codes, where codes starting with a lowercase letter indicate drug prescriptions (eAppendix 1). We included all Read codes with the first character ranging from “a” to “o” (Table 1) and clustered them based on their first 3 characters. For example, we considered “a136” (600 mg sodium bicarbonate) and “a137” (500 mg sodium bicarbonate) to be the same drug: “a13” (sodium bicarbonate). If the initial clustering produced nondescriptive clusters, we chose clustering based on the first 4 characters of the Read code instead. For example, we used the specific “dia7” (co-codamol) instead of the generic “dia” (compound analgesics A–L). Clustering resulted in a total of 2,023 drugs. We only included drugs that were prescribed in at least 2% of our study population to have sufficient statistical power to reliably identify associations between drug exposure and aSAH.

**Table 1:** Clustering Read Codes and Commonly Prescribed Drugs in Wales Between 2000 and 2019 Using the SAIL Databank

Term	Read code	Clustered drugs	Commonly prescribed^a^
Gastrointestinal drugs	a…	108	19
Cardiovascular drugs	b…	223	27
Respiratory drugs	c…	110	18
CNS drugs	d…	283	29
Drugs used in infections	e…	204	18
Endocrine drugs	f…	149	9
OBS/GYN/UTI drugs	g…	103	7
Chemotherapy/immunosuppressants drugs	h…	160	0
Hematology/dietetic drugs	i…	172	6
Musculoskeletal drugs	j…	75	14
Eye drugs	k…	109	8
ENT drugs	l…	71	10
Skin drugs	m…	190	32
Immunology drugs	n…	35	6
Anesthetic drugs	o…	31	2
Total		2,023	205

Abbreviations: ENT = ear, nose, and throat; OBS/GYN/UTI = obstetrics, gynecologic and urinary tract infection; SAIL = Secure Anonymised Information Linkage.

a Prescribed for at least 2% of patients within the case-control cohort.

**Figure 2:**
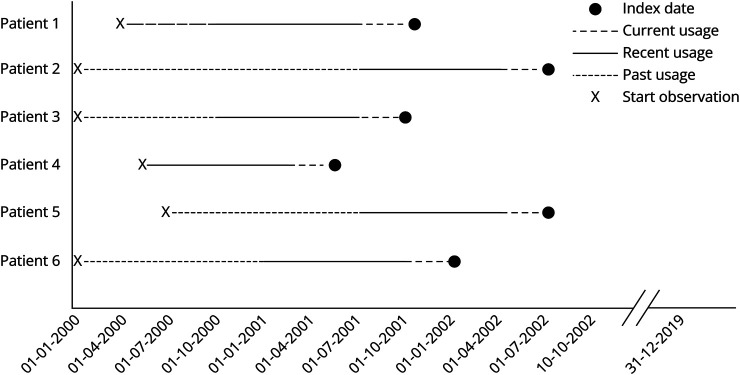
The Definition of 3 Nonoverlapping Time Periods to Capture the Current, Recent, and Past Effects of Drug Use on Aneurysmal Subarachnoid Hemorrhage Incidence Time periods are defined as follows: current (within 3 months before index date), recent (between 1 year before index date and 3 months before index date), and past (between January 1, 2000, and 1 year before index date).

We assessed drug exposure over 3 nonoverlapping time periods: current (within 3 months before index date), recent (between 1 year before index date and 3 months before index date), and past (between January 1, 2000, and 1 year before index date). This method was applied to capture a comprehensive view of how drug exposure influences aSAH incidence across temporal contexts (Figure 2), revealing short-term and long-term effects that might not be apparent within a single time period. We iterated through each commonly prescribed drug and mapped the patient's most recent drug prescription date (i.e., closest to their index date) to 1 of these 3 windows. We considered a patient to have never used the drug if they did not obtain a prescription for the drug or if the prescription date occurred after their index date.

### Covariates

To adjust for confounders, we considered routinely available covariates that may be related to both aSAH and drug exposure. These included smoking status, hypertension, alcohol abuse, body mass index (BMI), and an overall comorbidity score. In addition, we included health care utilization and socioeconomic status to address healthy user bias. We assessed health care utilization by counting the number of visits to a general practitioner in the year preceding the index date. For socioeconomic status, we assessed the Welsh Index of Multiple Deprivation (WIMD) of a patient's area of residence. We used code lists to cluster Read codes to define smoking status (defined either current, former, or never), hypertension (based on a general practitioner [GP] diagnosis), and alcohol abuse. Each of these variables was measured as close to the index date as possible and kept constant throughout the analysis. For BMI, we retrieved the most recent measurement before the index date. We used multivariate imputation to approximate BMI values where BMI data were missing. Similarly, for missing smoking status, we assumed “never.” Other categorical variables with missing values, such as hypertension, were imputed with a value of 0 to indicate the condition's absence. As a measure of overall comorbidity, we used Read-based diagnoses codes to define a modified version of the Elixhauser score,^16^ in which already incorporated covariates (i.e., hypertension, alcohol abuse, and obesity) were excluded. All analyses were performed in Python.^17^

### Statistical Analysis

We developed a binomial logistic regression model for each commonly prescribed drug with aSAH as the outcome. The model included drug exposure as a nominal variable with 4 levels (current, recent, past, never), with “never” acting as the reference category. All potential confounders described above were included as covariates in the model. For smoking, “never smoked” acted as the reference category. We calculated odds ratios (ORs) with 95% CIs for each commonly prescribed drug and applied Bonferroni correction to account for the number of commonly prescribed drugs tested. Following reviewer comments, we conducted a post hoc analysis in which we controlled for 2 additional covariates: a history of depression and a history of anxiety.

### Standard Protocol Approvals, Registrations, and Patient Consents

Ethical approval was not required because the study used only anonymized data. Approval was granted by the Information Governance Review Panel (IGRP, application number: 1261). Composed of government, regulatory, and professional agencies, the IGRP oversees and approves applications to use the SAIL databank.

### Data Availability

All anonymized linked health records used in this study are available through SAIL.

## Results

We identified 4,879 aSAH cases and matched them to 43,911 controls without aSAH. While our study sample was balanced at the index date in terms of sex (61.2% women in both cases and controls) and mean age (61.4, SD 15.4), aSAH cases visited their GP more frequently than controls (mean visits: 23 vs 19, respectively) (Table 2). In addition, more aSAH cases were current smokers (37% vs. 21%) or had a history of hypertension (42% vs. 37%) before the index date. Despite this, the mean modified Elixhauser score for cases was only marginally higher than for controls (1.8 vs 1.4).

**Table 2:** Baseline Characteristics of Cases of Aneurismal Subarachnoid Hemorrhage and Controls in Wales Between 2000 and 2019

	Cases	Controls
N	4,879	43,911
Matching criteria		
Observation period, d, median (range)	3,554 (365–7,304)	3,550 (365–7,304)
Age,^a^ mean (SD)	61.4 (15.4)	61.4 (15.4)
Female,^a^ n (%)	2,988 (61.2)	26,892 (61.2)
Smoking status, n (%)		
Current	1,787 (37)	9,005 (21)
Former	978 (20)	9,511 (22)
Never	1,840 (38)	22,413 (51)
Other		
Hypertension, n (%)	2,030 (42)	16,335 (37)
No. of general practitioner consultations, mean (SD)	23.3 (20.0)	18.9 (17.4)
Deprivation index, mean (SD)	22.4 (15.7)	20.9 (15.1)
Alcohol abuse, n (%)	333 (7)	1,784 (4)
BMI, mean (SD)	26.9 (5.6)	27.6 (5.8)
Modified Elixhauser score, mean (SD)	1.8 (1.7)	1.4 (1.6)

Abbreviation: BMI = body mass index.

a Matching criteria for nested-case control. Reported values at baseline (i.e., index date).

**Figure 3:**
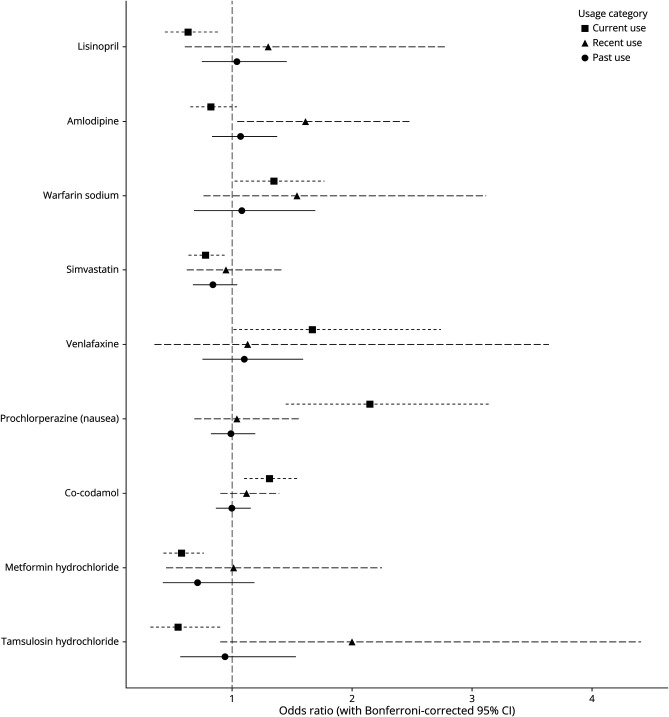
Commonly Prescribed Drugs Associated With aSAH Incidence Stratified by Recency of Use and in Comparison With Nonuse of the Same Drug aSAH = aneurysmal subarachnoid hemorrhage.

Clustering resulted in 2,023 unique drugs, of which 205 (10.1%) were commonly prescribed (Table 1). A total of 9 drugs had a statistically significant association with aSAH after Bonferroni correction (Figure 3, Table 3). An overview of all tested drugs and their corresponding statistics can be found in eTable 1.

**Table 3:** Drugs Associated With a Changed Incidence of Aneurysmal Subarachnoid Hemorrhage Incidence Using a Binomial Logistic Regression Model and Bonferroni Corrected 95% CIs for Use vs Nonuse of the Same Drug

Read code	Drug name	Current use OR (95% CI)	Recent use OR (95% CI)	Past use OR (95% CI)
Decreased incidence				
bi3	Lisinopril	0.63^a^ (0.44–0.90)	1.30 (0.61–2.78)	1.04 (0.75–1.45)
bxd	Simvastatin	0.78^a^ (0.64–0.96)	0.95 (0.62–1.45)	0.84 (0.68–1.04)
f41	Metformin	0.58^a^ (0.43–0.78)	1.01 (0.45–2.25)	0.71 (0.43–1.18)
gc7	Tamsulosin	0.55^a^ (0.32–0.93)	2.0 (0.90–4.43)	0.94 (0.58–1.53)
Increased incidence				
blb	Amlodipine	0.82 (0.65–1.04)	1.61^a^ (1.04–2.48)	1.07 (0.84–1.37)
bs1	Warfarin	1.35^a^ (1.02–1.79)	1.54 (0.76–3.13)	1.08 (0.69–1.69)
da7	Venlafaxine	1.67^a^ (1.01–2.75)	1.13 (0.35–3.64)	1.1 (0.76–1.59)
dhe	Prochlorperazine [nausea]	2.15^a^ (1.45–3.18)	1.04 (0.68–1.58)	0.99 (0.83–1.19)
dia2	Co-codamol	1.31^a^ (1.10–1.56)	1.12 (0.90–1.4)	1.00 (0.87–1.15)

Abbreviation: OR = odds ratio.

a Statistically significant (*p* < 0.05) after Bonferroni correction. Time windows are defined as current (within 3 months before index date), recent (between 1 year before index date and 3 months before index date), and past (between January 1, 2000, and 1 year before index date).

We found a significant decrease in aSAH incidence for current use of lisinopril (OR 0.63, 95% CI 0.44–0.90, Figure 3) vs nonuse, with a trend toward an increased risk in recent use (OR 1.30, 95% CI 0.61–2.78). We found a similar pattern for amlodipine, where there was a marginally nonsignificant decrease in aSAH incidence for current use (OR 0.82, 95% CI 0.65–1.04) vs nonuse, with an increased risk in recent use (OR 1.61, 95% CI 1.04–2.48). Notably, we did not find this trend for other antihypertensives (eTable 1).

Current use of 3 additional drugs was associated with a decreased risk of aSAH. Specifically, we found a reduced incidence of aSAH in current users of simvastatin (OR 0.78, 95% CI 0.64–0.96), metformin (OR 0.58, 95% CI 0.43–0.78), and tamsulosin (OR 0.55, 95% CI 0.32–0.93). No statistically significant associations were found between these drugs and aSAH in recent or past users. Notably, other drugs within the classes of ACE inhibitors, statins, antidiabetics, and α-blockers did not show significant associations with aSAH (eTable 1).

Conversely, current use of 4 other drugs was associated with an increased risk of aSAH. Specifically, we found an increased incidence of aSAH in current users of warfarin (OR 1.35, 95% CI 1.02–1.79), venlafaxine (OR 1.67, 95% CI 1.01–2.75), prochlorperazine (OR 2.15, 95% CI 1.45–3.18), and co-codamol (OR 1.31, 95% CI 1.10–1.56). No statistically significant associations were found between these drugs and aSAH in recent or past users. Other drugs within the drug classes of vitamin K antagonists, serotonin reuptake inhibitors, conventional antipsychotics, and compound analgesics did not show an association with aSAH.

The post hoc analyses in which we controlled for a history of depression and anxiety yielded no significant changes to the effects described above (eTables 2 and 3).

### Classification of Evidence

This study provides Class III evidence that some commonly prescribed drugs are associated with subsequent development of aSAH.

## Discussion

In this DWAS, we found 9 drugs that were associated with aSAH incidence. We found a similar trend for lisinopril and amlodipine, with a decreased aSAH risk in current use and an increased aSAH risk in recent use. Three additional drugs were associated with a decreased incidence of aSAH: simvastatin, metformin, and tamsulosin. By contrast, we found an increased incidence of aSAH in recent users of amlodipine and current users of warfarin, venlafaxine, prochlorperazine, and co-codamol.

We found a similar trend for lisinopril (an ACE inhibitor) and amlodipine (a calcium channel blocker), with increased aSAH incidence in recent use and decreased aSAH risk in current use (Figure 3). Other studies, while not unanimous,^18^ generally found a protective effect of antihypertensive use on aSAH incidence and outcome.^19-21^ However, the majority of these studies were cross-sectional and could therefore not distinguish between current and recent use. Our findings indicate a trend, with current antihypertensive use associated with a lower risk of aSAH (significant for lisinopril but not for amlodipine) and recent antihypertensive use associated with an increased risk of aSAH (significant for amlodipine but not lisinopril). An increased aSAH risk in recent use of antihypertensives may be due to confounding by indication, specifically caused by hypertension.^22^ However, we corrected for hypertension in our analysis, and confounding by indication would not explain a protective effect in current use. Alternatively, the increased aSAH risk observed in recent use may be related to drug treatment discontinuation (e.g., resistant hypertension, side effects, or contraindications). By contrast, a lower incidence in current use may be due to the antihypertensive's immediate effects, such as lowering blood pressure, or inhibiting the local renin-angiotensin system.^23^ Why we found this trend for lisinopril and amlodipine, but not for other antihypertensives remains an open question for further research.

We found a reduced aSAH incidence for current users of simvastatin (a statin) and metformin (an antidiabetic). Statins have been associated with a decreased incidence of aSAH in general, although the evidence is not consistent.^18,24,25^ A Dutch case-control study found that current statin use was associated with a lower risk of SAH (OR 0.77, 95% CI 0.55–1.07), whereas recent statin withdrawal was associated with an increased risk of SAH when compared with continued use (OR 2.34, 95% CI 1.35–4.05).^18^ Simvastatin may reduce the risk of aSAH by improving endothelial functions or by its anti-inflammatory effects on vascular walls.^26^ Antidiabetics, similar to statins, have been shown to reduce the risk of aSAH.^27^ Antidiabetics may lower aSAH risk by reducing the vascular complications associated with hyperglycaemia (e.g., endothelial damage, cerebral tight junction protein expression).^28,29^ Alternatively, our results with simvastatin and metformin could be explained by previously reported protective effects of hypercholesterolemia and diabetes on aSAH.^22^ However, we found no statistically significant results for other drugs that affect hypercholesterolemia or diabetes, implying that the lower incidence of aSAH in current simvastatin and metformin users may be drug-specific.

Finally, we found a reduced incidence of aSAH in current tamsulosin users. Specifically, we found a trend similar to that found for lisinopril and amlodipine, with an increased risk in recent use and a decreased risk in current use. Alpha blockers have not been studied for their association with aSAH, although we can speculate that alpha blockers affect aSAH risk by altering blood pressure or local vasodilation.^30^ It is currently unclear why we found an increased aSAH incidence in recent tamsulosin users, given that indications for tamsulosin prescription (e.g., prostatic hyperplasia, chronic prostatitis) are not currently known risk factors of aSAH. Future studies should further investigate these signals.

We found an increased risk of aSAH in current users of warfarin, an anticoagulant that functions by antagonizing vitamin K. Although the relationship between aSAH and warfarin has not been studied specifically, vitamin K antagonists in general have been linked to an increased risk of aSAH.^31,32^ As warfarin is known to elevate bleeding risk,^33^ it may consequently heighten the risk of aSAH. It is unknown whether warfarin causes an aneurysm to burst directly or indirectly by inducing tearing in the aneurysm wall. In this DWAS, detecting an increased risk associated with current use of warfarin can serve as a “positive control,” validating our methods and lending credibility to our other findings.

We additionally found an increased risk of aSAH in current users of venlafaxine (a serotonin and norepinephrine reuptake inhibitor, SNRI) and prochlorperazine (a conventional antipsychotic). SNRIs have, to the best of our knowledge, not been studied for their association with aSAH. SNRIs work by increasing noradrenergic activity,^3^ which can raise blood pressure and heart rate,^34,35^ potentially increasing the risk of aSAH. In addition, selective serotonin reuptake inhibitors, which are functionally similar to SNRIs, are known to increase the risk of bleeding.^36^ Prochlorperazine's effects on aSAH have not been studied before and require further investigation. A recently proposed relationship between psychiatric diseases and aSAH could potentially explain our findings for both venlafaxine and prochlorperazine.^37^ However, such residual confounding would not explain why we were unable to find an association for aSAH and other antidepressants and antipsychotics. Furthermore, we found that the association between aSAH and both venlafaxine and prochlorperazine remained when controlling for a history of depression and anxiety. This suggests that the observed relationships were likely due to the drugs themselves rather than underlying psychiatric diseases.

Finally, we found an increased risk of aSAH in current users of co-codamol, a compound analgesic consisting of codeine and paracetamol. We were unable to investigate the individual effects of each active substance because of limitations in our clustering of read codes. Thus, the observed association could be attributed to the use of either of these medications or a synergistic effect of their concomitant use. The relationship between aSAH and co-codamol has not been studied before, and their mechanisms (e.g., blood pressure changes, vasodilation) require further investigation.

This study had several strengths. First, identifying nearly 5,000 aSAH cases from a large nationwide data set, and focusing solely on commonly prescribed drugs, increased our statistical power to detect even relatively subtle effects. Second, we were able to differentiate acute from chronic effects by examining recency of use with nonoverlapping windows. Finally, we mitigated healthy-user bias and confounding by adjusting for health care utilization and a variety of characteristics related to overall health and aSAH. Although we used records from an EHD, where data was not primarily recorded for research use, the SAIL databank has a reputation for good coverage and linkage.^38^ For instance, only 6% of individuals in the SAIL databank lacked smoking status data.

This study also had several limitations. First, observed findings may be due to associations between the indications and aSAH (i.e., confounding by indication). For example, a lower incidence of aSAH in metformin users may simply reflect a lower incidence of aSAH in diabetics. However, we aimed to reduce confounding by indication by controlling for known aSAH risk factors. Second, we assumed that a drug prescription translates to proper drug use. In reality, however, a patient may not take their drugs or use them incorrectly, which results in exposure misclassification. Thus, our results could be skewed if the discrepancy between prescribed and actual drug use differs between cases and controls. Third, by clustering the drugs investigated in our study, we lost the specificity provided by a Read code. As a result, we were unable to study dose-response relationships, but were able to study drug-specific (rather than class-specific) effects. Fourth, at this stage we were unable to study duration of use of each medication and differentiate between first-time and long-term users. In addition, drug exposure windows were defined identically for each drug, ignoring drug-specific duration of effects. We thus may have missed differences between acute and long-term effects of drugs on aSAH. Another limitation relates to the definition of aSAH. We chose to include *ICD-10* codes I60.8 (“other nontraumatic subarachnoid hemorrhage”) and I60.9 (“nontraumatic subarachnoid hemorrhage, unspecified”) because misclassification can occur when using ICD codes in general^39^ and for aSAH specifically.^40^ By including these codes, we may have included nonaneurysmal subarachnoid hemorrhage cases, potentially introducing outcome misclassification. However, nonaneurysmal subarachnoid hemorrhage cases are relatively rare,^1^ and we decided that the potential cost to specificity was worth the increase in sample size and sensitivity, which was in line with our primary objective of hypothesis generation.

In conclusion, our results suggest that 9 drugs may be associated with aSAH, where current use of lisinopril, simvastatin, metformin, tamsulosin, and potentially amlodipine showed a decreased risk. Using these signals as a starting point, future research should use a more hypothesis-driven approach to further investigate these associations and differentiate between drug class and specific drug substance effects. In addition, this research may help identify additional risk factors for aSAH, potentially leading to new pharmacologic therapy options for aneurysmal management.

## Appendix Authors

**Table:**

Name	Location	Contribution
Jos P. Kanning, MSc	UMC Utrecht Brain Center, Department of Neurology and Neurosurgery, and Julius Center for Health Sciences and Primary Care, University Medical Center Utrecht, Utrecht University, the Netherlands	Drafting/revision of the manuscript for content, including medical writing for content; study concept or design; analysis or interpretation of data
Shahab Abtahi, PhD	Division of Pharmacoepidemiology and Clinical Pharmacology, Utrecht Institute for Pharmaceutical Sciences, Utrecht University, the Netherlands	Drafting/revision of the manuscript for content, including medical writing for content; study concept or design; analysis or interpretation of data
Christian Schnier, PhD	Infection Medicine, Edinburgh Medical School, The University of Edinburgh, United Kingdom	Drafting/revision of the manuscript for content, including medical writing for content; major role in the acquisition of data; study concept or design; analysis or interpretation of data
Olaf H. Klungel, MD	Julius Center for Health Sciences and Primary Care, University Medical Center Utrecht, and Division of Pharmacoepidemiology and Clinical Pharmacology, Utrecht Institute for Pharmaceutical Sciences, Utrecht University, the Netherlands	Drafting/revision of the manuscript for content, including medical writing for content; study concept or design; analysis or interpretation of data
Mirjam I. Geerlings	Julius Center for Health Sciences and Primary Care, University Medical Center Utrecht, Utrecht University; Department of General Practice, Amsterdam UMC, location University of Amsterdam; Amsterdam Public Health, Aging & Later Life, and Personalized Medicine; Amsterdam Neuroscience, Neurodegeneration, and Mood, Anxiety, Psychosis, Stress, and Sleep, the Netherlands	Drafting/revision of the manuscript for content, including medical writing for content; study concept or design; analysis or interpretation of data
Ynte M. Ruigrok, MD	UMC Utrecht Brain Center, Department of Neurology and Neurosurgery, University Medical Center Utrecht, the Netherlands	Drafting/revision of the manuscript for content, including medical writing for content; major role in the acquisition of data; study concept or design; analysis or interpretation of data

## Glossary

aSAH: Aneurysmal subarachnoid hemorrhage
BMI: body mass index
DWAS: drug-wide association study
EHD: electronic health care database
GP: general practitioner
*ICD-9*: *International Classification of Diseases, Ninth Revision*
*ICD-10*: *International Classification of Diseases, Tenth Revision*
IGRP: Information Governance Review Panel
OR: odds ratio
SAIL: Secure Anonymised Information Linkage
SNRI: serotonin and norepinephrine reuptake inhibitor
